# Rare solid tumors in a patient with Wiskott–Aldrich syndrome after hematopoietic stem cell transplantation: case report and review of literature

**DOI:** 10.3389/fimmu.2023.1229674

**Published:** 2023-09-13

**Authors:** Emma Coppola, Giuliana Giardino, Massimo Abate, Francesco Paolo Tambaro, Delfina Bifano, Elisabetta Toriello, Antonio De Rosa, Francesca Cillo, Claudio Pignata, Emilia Cirillo

**Affiliations:** ^1^ Department of Translational Medical Sciences, Pediatrics Section, Federico II University of Naples, Naples, Italy; ^2^ Pediatric Oncology Department, Santobono-Pausilipon Children’s Hospital, Naples, Italy; ^3^ Division of Stem Cell Transplantation and Cell Therapy, Pediatric Oncology Department, Santobono-Pausilipon Children’s Hospital, Naples, Italy; ^4^ Department of Pathology, Santobono-Pausilipon Children’s Hospital, Naples, Italy

**Keywords:** Wiskott Aldrich syndrome, inborn errors of immune system, malignancies, hematopoietic stem cell transplantation, hemangioendothelioma kaposiform desmoid tumor, case report

## Abstract

**Background and aims:**

Wiskott–Aldrich syndrome (WAS) is an X-linked recessive primary immunodeficiency disorder characterized by severe eczema, recurrent infections, and micro-thrombocytopenia. Allogeneic hematopoietic stem cell transplantation (HSCT) is a potentially curative therapeutic option for patients with classic form. The risk of developing post-transplant tumors appears to be higher in patients with WAS than in other inborn errors of immunity (IEIs), but the actual incidence is not well defined, due to the scarcity of published data.

**Methods:**

Herein, we describe a 10-year-old patient diagnosed with WAS, treated with HSCT in the first year of life, who subsequently developed two rare solid tumors, kaposiform hemangioendothelioma and desmoid tumor. A review of the literature on post-HSCT tumors in WAS patients has been performed.

**Results:**

The patient received diagnosis of classic WAS at the age of 2 months (Zhu score = 3), confirmed by *WAS* gene sequencing, which detected the nonsense hemizygous c.37C>T (Arg13X) mutation. At 9 months, patient underwent HSCT from a matched unrelated donor with an adequate immune reconstitution, characterized by normal lymphocyte subpopulations and mitogen proliferation tests. Platelet count significantly increased, even though platelet count never reached reference values. A mixed chimerism was also detected, with a residual WASP− population on monocytes (27.3%). The patient developed a kaposiform hemangioendothelioma at the age of 5. A second abdominal tumor was identified, histologically classified as a desmoid tumor when he reached the age of 10 years. Both hematopoietic and solid tumors were identified in long-term WAS survivors after HSCT.

**Conclusion:**

Here, we describe the case of a patient with WAS who developed two rare solid tumors after HSCT. An active surveillance program for the risk of tumors is necessary in the long-term follow-up of post-HSCT WAS patients.

## Introduction

Wiskott–Aldrich syndrome (WAS) is a rare X-linked recessive inborn error of immune system (IEI) characterized by severe eczema, recurrent infections, and thrombocytopenia with small platelets (1). The *WAS* gene, located on the short arm of the X chromosome, encodes the WAS protein (WASp), which is expressed only in non-erythroid hematopoietic cells. WASp plays a crucial role in cell surface signaling to the cytoskeleton and is involved in actin polymerization; its loss of function impairs basic and necessary functions of the cells of the immune system, such as migration and tissue localization, adhesion, phagocytosis, and cell–cell interaction, resulting in failure of the antigen presentation system and immunological synapse assembly (2). The impaired functioning of the immune system, on the one hand, predisposes to severe and recurrent infections; on the other hand, it increases susceptibility to autoimmune manifestations and tumors. A potentially curative therapeutic option for patients with classic form is represented by allogeneic hematopoietic stem cell transplantation (HSCT) (3, 4), with an estimated 3-year survival from transplantation of approximately 88% and better overall survival for patients aged <5 years (5–7). Patients lacking a matched donor may benefit from *ex vivo* lentiviral hematopoietic stem and progenitor cell gene therapy (HSPC-GT) (8).

A risk of developing post-transplant tumors has been documented in IEIs patients and estimated to be higher in WAS patients than in other genetic forms of IEI, but the actual incidence is unknown. The most frequently reported tumors are lymphoproliferative disorders, especially EBV-related disorders. From the available data, only six cases of solid tumors have been reported in the post-transplant period, even in long-term observation.

Herein, we described a 10-year-old patient diagnosed with WAS and undergoing HSCT in the first year of life, who subsequently developed two rare solid tumors, kaposiform hemangioendothelioma and desmoid tumor (9), https://www.ncbi.nlm.nih.gov/pmc/articles/PMC8167394/). Furthermore, we used PubMed to search for all the studies published over the last 40 years using the keywords, namely, “Wiskott–Aldrich syndrome” and “cancer” or “tumor” or “malignancy” and “HSCT” in order to perform a literature review of post-HSCT malignancies in WAS patients.

## Case report

The patient was born to a healthy non-consanguineous parents. At the age of 15 days, he was hospitalized due to fever, diarrhea, and dehydration. Blood tests showed an increase in inflammatory markers and thrombocytopenia (7,000/mm^3^). Broad-spectrum antibiotic therapy was started for suspected sepsis, and platelet transfusion was performed with little increase in platelet count (25,000/mm^3^). Intravenous immunoglobulin (800mg/kg) infusion was administered, with no improvement in platelet count. Hypereosinophilia (4,680/mm^3^) and an increase in IgE (>5,000 IU/L) were identified. Bone marrow aspirate showed a reduced number of megakaryocytes. A diagnosis of WAS was hypothesized due to the low mean platelet volume of 6 fl. The flow cytometric expression of the WASp protein was reduced on platelets, monocytes, and lymphocytes (17%), and *WAS* gene sequencing detected a previously reported c.37C>T (Arg13X) nonsense hemizygous mutation in exon 1 (10, 11), thereby confirming the diagnosis. An extensive immunological workup was performed through the evaluation of lymphocyte proliferation to mitogens, which showed a normal response to phytohemagglutinin, concanavalin A, pokeweed, and phorbol myristate acetate mitogens, but an abnormal response to the CD3 cross-linking. Lymphocyte subpopulations showed a reduction in all subclasses (CD3+, 41%; absolute count, 907 cells/mm^3^; CD4+, 30%; absolute count, 663 cells/mm^3^; CD8+, 6%; absolute count, 132 cells/mm^3^; CD19+, 2%; absolute count, 44 cells/mm^3^). Normal serum levels of immunoglobulins were detected.

At the age of 4 months, he developed eczema in the face and auricles. During the follow-up, he suffered from recurrent lower airway infections, mainly due to *Klebsiella pneumoniae* infection. According to the clinical score proposed by Zhu et al., the phenotype was suggestive of a classic form of WAS (total score = 3), and the patient was considered eligible for HSCT. At 9 months, the patient underwent HSCT from a matched unrelated donor. Conditioning regimen consisted of intravenous Treosulfan 14 g/m^2^/daily for 3 days (day 7–6–5), Fludarabine 40 mg/m^2^/daily for 4 days (day 7–6–5–4), and antithymocyte globulin (Thymoglobulin^®^) 2 mg/kg/daily for 3 days (day 4–3–2). Four days after stem cells infusion, he developed group D *Streptococcus* lung infection treated with intravenous teicoplanin. *Pseudomonas aeruginosa* pneumonia was documented 10 months after transplantation

After transplantation, an adequate immune reconstitution was obtained, characterized by normal lymphocyte subpopulations and mitogen proliferation tests, with a normal anti-CD3 response. The platelet count increased significantly, but remained below the normal reference range, not exceeding 100,000/mm^3^, and with a fluctuating mean platelet volume (range, 5.5–10 fl). No bleeding events were reported. A mixed chimerism was also highlighted at the age of 5 years, with the following percentages of WASP+ cells (donor cells) in the leukocyte populations analyzed through flow cytometry: total T lymphocytes, 99.2%; CD3+CD4+, 99%; CD3+CD8+, 99.4%; B lymphocytes, 93.8%; NK cells, 95.1%; and monocytes, 72.7%.

**Figure 1:**
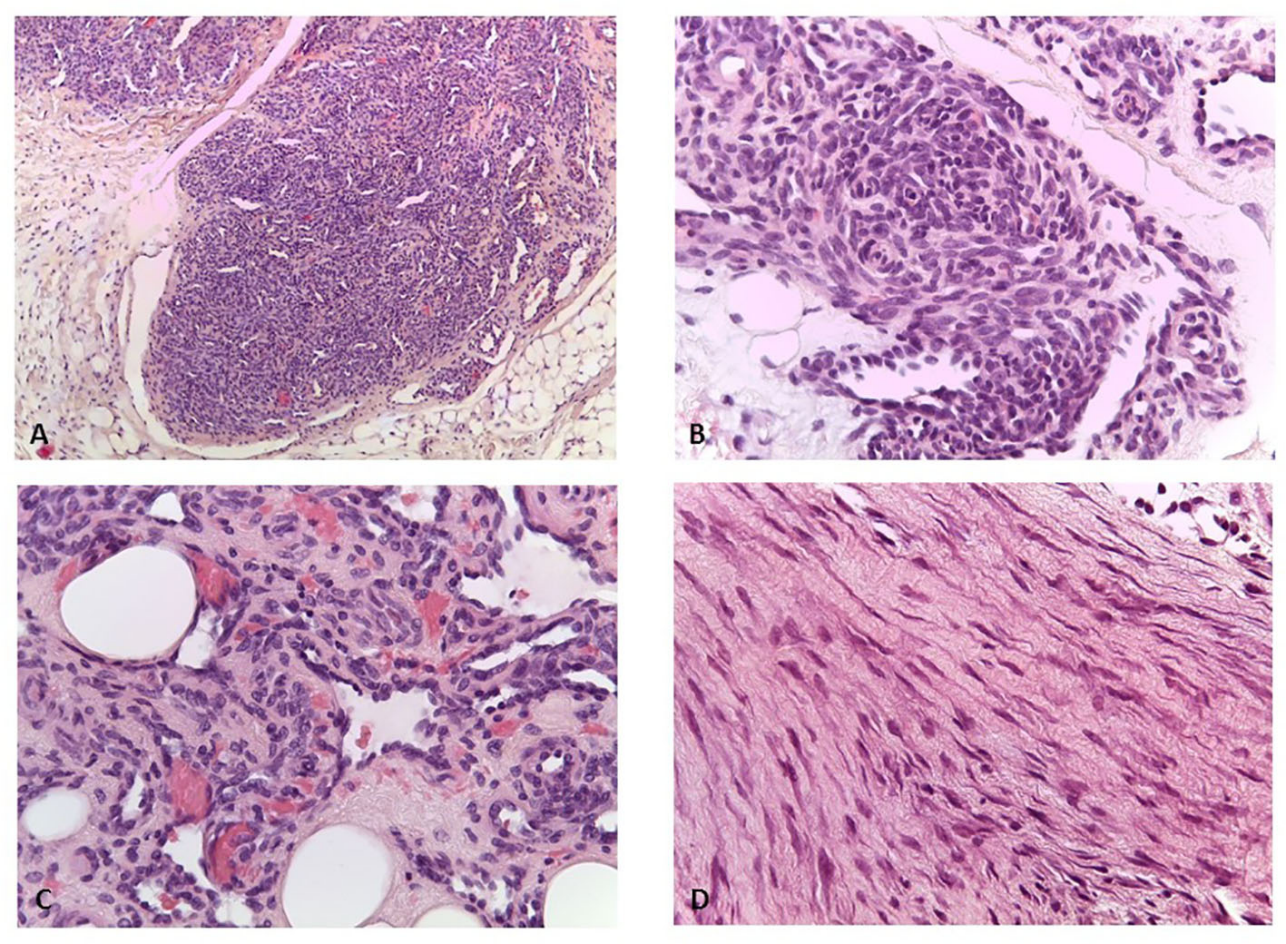
**(A)** Kaposiform emangioendothelioma: lobules of tumor cells coalesce and the capillaries form solid sheets with occasional slit-like lumens (H&E, ×100). **(B)** Kaposiform hemangioendothelioma: in some areas, the endothelial cells have a prominent spindled appearance (H&E, ×200). **(C)** Kaposiform hemangiothelioma: lumens filled with red blood cells (H&E, ×400). **(D)** Desmoid fibromatosis: spindled fibroblasts are arranged in bundles (H&E, ×400).

The patient had no other clinically significant events until the age of 5, when a small, painless, movable nodular formation in the left abdominal wall was evident. The ultrasound showed a nodular formation of 16×9 mm of probable angiomatous nature. The lesion was surgically removed, and histological examination was suggestive of kaposiform hemangioendothelioma. Histology of the vascular lesion showed irregular tumor nodules, characterized by spindle-shaped endothelial cells and peripheral ectatic slit-like vascular channels. Tumor cells showed uniform hyperchromatic spindled nuclei arranged in short fascicles with slit-like vascular lumina surrounded by a dense collagenous stroma (**Figures 1A–C**). Immunohistochemistry was positive for CD31, CD34, and podoplanin (D2–40), and negative for GLUT1. A second surgical revision was performed to widen the excision of the margins. PET-TC total body excluded distant metastatic dissemination.

**Figure 2:**
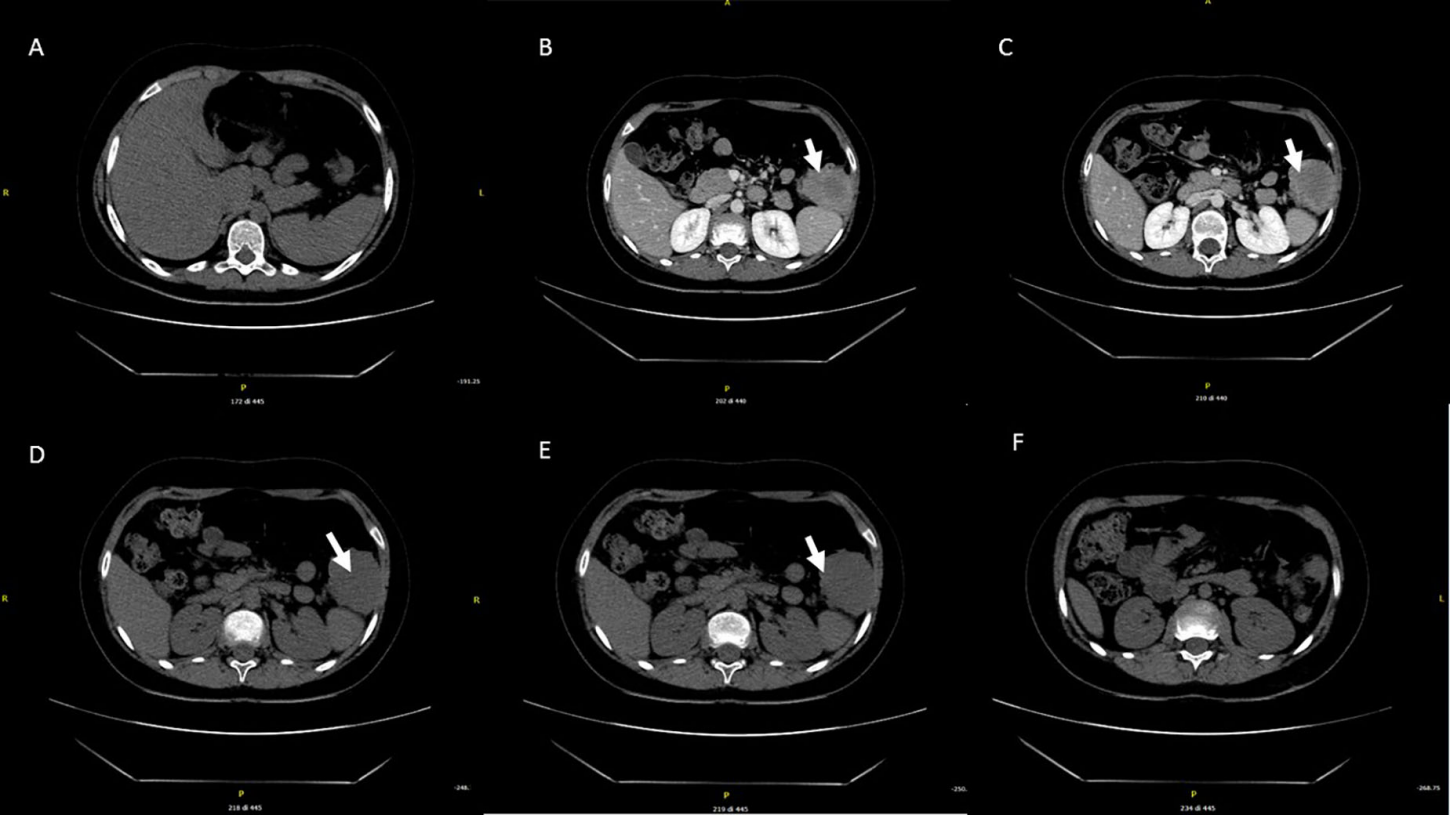
Abdominal CT performed at the diagnosis. The expansive mass is located in the left hypochondrium, inseparable from the contiguous walls of the colon and in close proximity to the lower pole of the spleen **(A–F)**.

At age 10, an intra-abdominal mass was identified by ultrasound during periodic follow-up. The mass was caudal and contiguous to the inferior pole of the spleen, with irregular margins, hypoechoic, approximately 55 × 50 mm, not vascularized on color Doppler. Abdomen CT scan showed that the expansive formation occupied the splenic flexure of the colon and part of the descending colon, was inseparable from the colonic walls, incorporating the left colonic artery and showed close contiguity with the spleen and pancreatic tail; it was also indissociable from the ipsilateral kidney and infiltrated the contiguous muscle planes (**Figures 2A–F**). For this reason, the patient was not referred for surgical treatment. Biopsy was performed and microscopic examination showed a uniform proliferation of spindle cells surrounded by a stroma of abundant collagen (**Figure 1D**). In immunohistochemistry, it was characterized by nuclear positivity for vimentin, being negative for desmin, S-100, h-caldesmon, CD34, and β-catenin. Vinorelbine and methotrexate chemotherapy was started for a total of 12 cycles. After this treatment, a reduction in mass volume was observed, with a longitudinal diameter of 46 mm and a maximum diameter on the transverse plane of 40 × 25 mm, although still bound to the spleen, the pancreas, and the intestine. The patient is currently off therapy but under close follow-up, to evaluate a possible progression of the tumor, which cannot be surgically removed due to its close proximity to the surrounding structures.

At the age of 10, STR-based chimerism analysis performed on whole blood showed mixed chimerism, with a percentage of donor cells equal to 66.3%.

**Figure 3:**
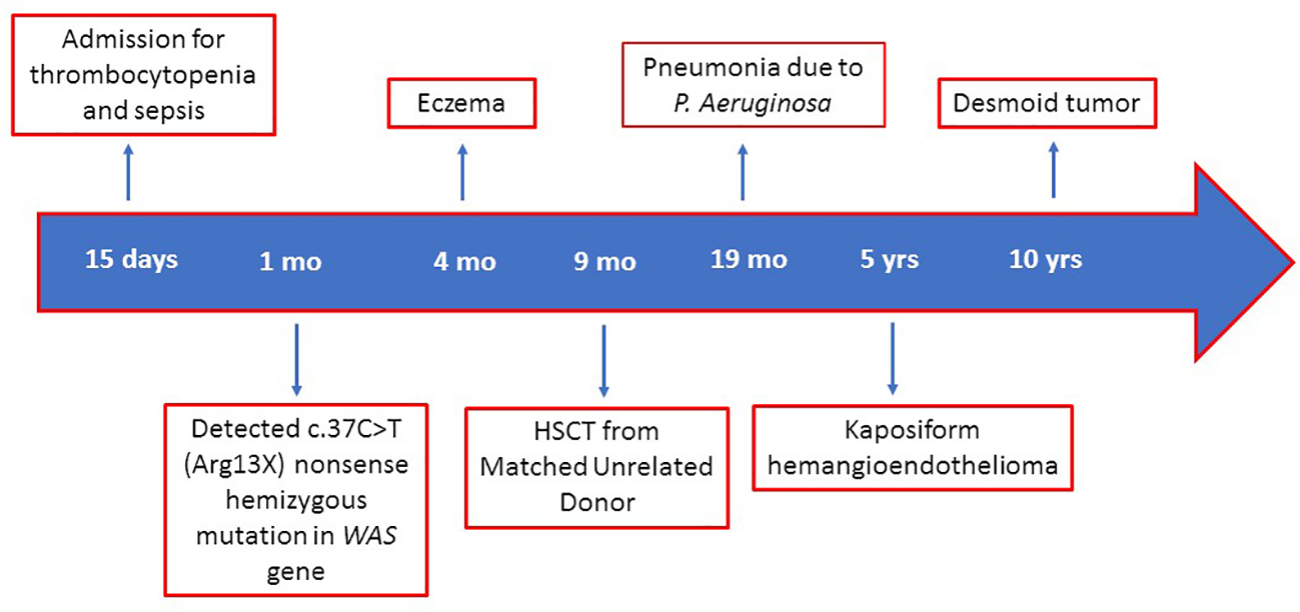
Timeline of major clinical events of the patient. HSCT, hematopoietic stem cells transplantation; WAS, Wiskott–Aldrich syndrome; mo, months; yrs, years.

The main events of the patient’s clinical history are summarized in **Figure 3**.

## Review of previously reported post-HSCT malignancies in WAS

To identify the previously reported WAS patients who developed post-HSCT malignancies, we searched PubMed for post-HSCT follow-up reports of patients diagnosed with IEI that included WAS patients. A total of 40 studies were identified. Among these, we selected only studies that made explicit mention of post-HSCT cancer surveillance in the follow-up. Another exclusion criterion was shorter post-HSCT follow-up (<10 years). However, excellent outcome following HSCT was reported in several studies even though they were not specifically directed to follow-up of secondary cancers (5, 12). Nine studies fulfilling this criterion were selected for the analysis (**Table 1**).

**Table 1:** Literature review on long-term follow-up post-HSCT and malignancies for WAS patients.

	Recruitment from–to	No. of WAS patients	No. WAS patients that develop tumor post HSTC	Hematological tumor (n)	Solid tumor (n)	Years of observation post transplant
Kamani et al., 2011 (13)	1968–2003	348	12	EBV-PTLD (3) MDS (2) PTLD (2) B-cell lymphoma (3) Large cell malignant lymphoma EBV+ (1) Immunoblastic BCL (1)	None (0)	15 y (1968–2003)
Filipovich et al., 2001 (14)	1968–1996	170	6	EBV-PTLD (3) PTLD (2) AML (1)	None (0)	28 y
Ozsahin et al., 2008 (15)	1979–2001	96	0	None (0)	None (0)	22 y
Moratto et al., 2011 (16)	1980–2009	195	5	EBV–PTLD (4)	Embryonal carcinoma of the testis (1)	40 y
Kobayashi et al., 2006 (17)	1985–2004	57	0	None (0)	None (0)	19 y
Shin et al., 2012 (18)	1990–2009	47	2	EBV-PTLD (2)	None (0)	19 y
Mitchell et al., 2013 (19)	1992–2008	27	0	None (0)	None (0)	16 y
Elfeky et al., 2018 (20)	1996–2016	34	2	EBV-PTLD (1)	SCC at gastrostomy site (1)	10 y
Golwala et al., 2023 (21)	2000–2018	34	4	None (0)	Osteochondroma (4)	18 y

EBV-PTLD, post-transplant Epstein–Barr virus-lymphoproliferative disease; MDS, myelodysplastic syndrome; PTLD, post-transplant lymphoproliferative disease; BCL, B-cell Lymphoma; AML, acute myeloid leukemia.

Kamani et al. collected a total of 2,266 patients with IEI who had undergone allogeneic HSCT between 1968 and 2003. Of the 2,266 patients, 360 had WAS (13). Among them, 12 patients (3.4% of the WAS cohort) developed post-transplant cancer, including post-transplant EBV-lymphoproliferative disease (EBV-PTLD) and B-cell lymphoma. Four patients (40%) underwent a haploidentical transplant, one received transplantation from matched related donor (RD), three from matched unrelated donor (MUD), and two from unrelated donor (UR). Five patients received a conditioning regimen with busulfan, cyclophosphamide, and anti-thymocyte globulin. Only two out of these patients were alive at the last follow-up. None of these patients developed a solid tumor. Elfeky et al. report the follow-up of 34 WAS patients who underwent HSCT between 1996 and 2016 (20). Two patients (5.9%) developed post-transplant cancer. One of the two patients, who received an HSCT from MUD, developed a squamous cell carcinoma at the gastrostomy site, 3 months after transplantation. More recently, the same group described in a cohort of 429 IEI patients who received HSCT between 2000 and 2018, four subjects (11.8% of the WAS cohort) who developed osteochondroma, a benign solid tumor of the bone. Of them, two patients showed mixed chimerism. Traditionally, this condition has been associated with the use of radiation therapy in malignancies, but none of the children who developed osteochondroma received radiotherapy treatment, suggesting the role of different factors in the pathogenesis of bone tumors (21, 22). In the Moratto et al. cohort, including 194 WAS patients who received HSCT, four subjects developed hematological malignancies, while a recurrence of a testicular carcinoma was observed in a further patient (16). Data from the other four studies are shown in the table (14, 18). No post-HSCT cancer cases were described in the study of Ozsahin et al. (15), Kobayashi et al. (17), and Mitchell et al. (19).

Overall, data relating to 966 WAS patients undergoing HSCT, excluding the Golwala et al. cohort, were reported from 1968 to 2018. Adding our patient to the literature series (n = 967), 3.3% of patients developed post-transplant cancer (n = 32, confidence interval (CI) 2.3%–4.6%) and only in 0.8% of cases a solid tumor (n = 8, CI 0.04–1.6%). A definitive analysis on post-transplant chimerism of patients who developed solid tumors could not be performed due to the paucity of published data.

## Discussion

Here, we described the case of a patient with WAS who developed two rare solid tumors after allogeneic HSCT. The first tumor, KE, was found 5 years after HSCT. KE is a rare tumor, currently classified as an intermediate (locally aggressive) vascular tumor (9), originating from endothelial cells, which occurs typically in childhood. KE is characterized by an infiltrative growth pattern, rare tendency to spontaneous regression, and possible distant metastasis. Resection is the treatment of the first choice. Life-threatening thrombocytopenia and consumptive coagulopathy, known as the Kasabach–Merritt phenomenon (KMP), have been described in patients with intrathoracic and retroperitoneal lesions (23). KMP was excluded in our patient because thrombocytopenia was not severe as usually seen in KMP and there was no worsening of the platelet count. The etiopathogenesis of KE and molecular aspects are still unclear, and unlike Kaposi sarcoma of childhood, it is not associated with human herpes virus-8 infection. Kaposi sarcoma of childhood, an inflammatory neoplasm of the endothelial cell, has previously been described in a few untreated WAS patients (24, 25). In one of these subjects, a complete remission from KS has not been achieved until HSCT was performed.

The second tumor was found 10 years after HSCT. It has been described as a desmoid tumor (DT), but does not fully reflect its immunohistochemical features. DT is a tumor that originates from mesenchymal tissues, with no tendency to metastasize but with a high recurrence rate. It is currently classified, according to WHO, as an intermediate (locally aggressive) fibroblastic/myofibroblastic tumor (9). There are two peaks of incidence among children and adults, one in the age group of 6–15 years and another from age of puberty to 40 years of age. The pathogenesis of DT seems to be linked to a dysfunction of the Wnt/β-catenin cascade, and there are two variants: the familial adenomatous polyposis (FAP)-related DT and the sporadic DT, most frequent, due to somatic mutation of *CTNNB1* gene. Histologically, DT is characterized by a poorly defined and uniform proliferation of spindle cells packaged within an abundant collagen stroma and a vascular network not surrounded by a capsule. In immunohistochemistry, it is positive for β-catenin, vimentin, Cox2, c-KIT, PDGFRb, androgen receptors, beta estrogen receptors and negative for desmin, S-100, h-caldesmon, CD34, and CKIT (26–28). The biopsy sample of our patient reflected these histological features, with spindle cells immersed in a stroma of abundant collagen. At immunohistochemistry, however, it was characterized by nuclear positivity for vimentin and being negative for β-catenin. Unfortunately, further molecular evaluations were not performed on the tumor sample due to the limited availability of biological tissue.

WAS is associated with higher susceptibility to autoimmunity and tumors (29). This susceptibility may persist in some patients even after HSCT. Despite that autoimmune manifestations have been frequently reported in WAS patients before and after HSCT, the pathogenesis of autoimmunity is not fully understood. Pre-transplant autoimmunity is not considered a risk factor for the development of post-transplant autoimmunity; however, WAS patients with pre-transplant autoimmunity are more prone to malignancy development (12, 30, 31). Risk factors for post-transplant autoimmunity appears to be donor type, with a higher incidence detected in matched unrelated donors, and mixed chimerism, especially on myeloid cells, although this trend does not appear to be confirmed in the long-term follow-up (12). While the incidence of autoimmunity is estimated to be 20% within the first 2 years after transplantation, the incidence of hematopoietic or solid tumors in WAS patients after transplantation is not well defined (32). Patients who underwent HSCT for reasons different from WAS had a risk of solid tumors significantly higher than the general population. Curtis et al. reported data on approximately 20,000 people undergoing HSCT, which did not include patients with IEIs. The study highlighted the existence of significant post-transplant risk of developing some types of solid cancers, such as connective tissue cancers (33). In particular, this study showed that the cumulative incidence rate of new solid cancers 5, 10, and 15 years after transplantation was 0.7% (95% CI, 0.4%–0.9%), 2.2% (95% CI, 1.5–3.0%), and 6.7% (95% CI, 3.7–9.6%), respectively; the corresponding values for the general population were 0.3%, 0.6%, and 0.8%.

Considering the total number of WAS patients who underwent transplantation in the last 50 years, the percentage of post-HSCT tumors is higher than the general population (3.3%), as reported by Kamani et al. However, the overall risk of solid cancer does not appear different from that observed in the general population. Almost all tumors observed in WAS patients during post-HSCT follow-up were hematological, such as lymphoproliferative disorders, EBV-related or not, and lymphomas. Among the six cases of solid tumors, not including our patient, two of them were malignant, a testicular tumor and a squamous cell carcinoma. In particular, the testicular cancer was a recurrence of a pre-transplant tumor, while the squamous cell carcinoma was described at the site of a gastrostomy.

Due to the limited data available, it is not possible to provide conclusive data on the correlation between the chimerism status and tumor development, since malignancies were reported in both patients with complete or mixed chimerism. It is possible to hypothesize that independent risk factors, such as the type of conditioning regimens or individual genetic predispositions, may be involved in the tumorigenesis. In patients with mixed chimerism, the persistence of WASp-deficient cells may impair the ability of the immune system to suppress reactive clones, as underlined by the higher incidence of hematological tumors (2, 31).

Our patient developed a first tumor originating from endothelial cells 5 years after transplantation and a second tumor originating from fibroblasts 10 years after transplantation. Interestingly, patient chimerism was highest on monocytes, with over 20% of recipient cells. Recent evidence shows that WAS− macrophages have a tendency to differentiate into a pro-inflammatory phenotype, with increased release of pro-inflammatory cytokines. In addition to pro-inflammatory cytokines, activated macrophages also secrete fibroblast growth factor and vascular endothelial growth factor (34). Recently, the role of monocytes macrophages as tumor-promoting inflammation has been described (35). It is possible that the abnormal activation of macrophages has induced the release of growth factors and the consequent proliferation of specific cells, which, when subjected to prolonged replicative stimulation, accumulated pro-oncogenic mutations. However, we cannot exclude an underlying genetic predisposition that determines a susceptibility to oncogenic transformation when exposed to abnormal proliferative stimuli.

In conclusion, both hematopoietic and solid tumors may occur in long-term survivors of allo-HSCT. Our analysis shows that the risk of solid tumors in post-HSCT WAS patients is comparable to that of the general population. Differently, the risk of hematological tumors is higher than in the general population, and this highlights the need to implement a long-term oncological follow-up for these patients. A limitation of the study is the exclusion of some cohorts, even numerous, due to the short follow-up and/or failure to mention the oncological follow-up. It cannot be excluded that tumors, even if not clearly explained, did not arise in these cohorts.

Further studies are also needed to evaluate oncological risk among this cohort of patients and understand the mechanism and risk factors, which would require a large prospective study.

## Patient perspective

Throughout the process, the patient and his parents were informed of treatment options and risks. They realized the complexity of the clinical condition and appreciated the multidisciplinary approach.

## Data availability statement

The original contributions presented in the study are included in the article/supplementary material. Further inquiries can be directed to the corresponding author.

## Ethics statement

Written informed consent was obtained from the patient’s parents for the publication of any potentially identifiable images or data included in this article.

## Author contributions

ECo, CP, and ECi conceived of the study and drafted the manuscript. DB participated in the histopathological evaluation. GG, MA, FT, ET, AR, and FC participated in acquisition of data and analysis. All authors contributed to the article and approved the submitted version.
